# Is hypofractionated whole pelvis radiotherapy (WPRT) as well tolerated as conventionally fractionated WPRT in prostate cancer patients? The HOPE trial

**DOI:** 10.1186/s12885-020-07490-0

**Published:** 2020-10-09

**Authors:** Lucas C. Mendez, Andrew J. Arifin, Glenn S. Bauman, Vikram M. Velker, Belal Ahmad, Michael Lock, Varagur M. Venkatesan, Tracy L. Sexton, George B. Rodrigues, Jeff Chen, Bryan Schaly, Andrew Warner, David P. D’Souza

**Affiliations:** 1 Division of Radiation Oncology, London Regional Cancer Program, 800 Commissioners Road East, London, Ontario N6A 5W9 Canada; 2 Department of Physics and Engineering, London Regional Cancer Program, 800 Commissioners Road East, London, Ontario N6A 5W9 Canada

**Keywords:** Prostate cancer, Brachytherapy, Radiotherapy, Pelvis, Hypofractionation, Toxicity, Quality of life, Cancer

## Abstract

**Background:**

Patients with high-risk prostate cancer are at increased risk of lymph node metastasis and are thought to benefit from whole pelvis radiotherapy (WPRT). There has been recent interest in the use of hypofractionated radiotherapy in treating prostate cancer. However, toxicity and cancer outcomes associated with hypofractionated WPRT are unclear at this time. This phase II study aims to investigate the impact in quality of life associated with hypofractionated WPRT compared to conventionally fractionated WPRT.

**Methods:**

Fifty-eight patients with unfavourable intermediate-, high- or very high-risk prostate cancer will be randomized in a 1:1 ratio between high-dose-rate brachytherapy (HDR-BT) + conventionally fractionated (45 Gy in 25 fractions) WPRT vs. HDR-BT + hypofractionated (25 Gy in 5 fractions) WPRT. Randomization will be performed with a permuted block design without stratification. The primary endpoint is late bowel toxicity and the secondary endpoints include acute and late urinary and sexual toxicity, acute bowel toxicity, biochemical failure-, androgen deprivation therapy-, metastasis- and prostate cancer-free survival of the hypofractionated arm compared to the conventionally fractionated arm.

**Discussion:**

To our knowledge, this is the first study to compare hypofractionated WPRT to conventionally fractionated WPRT with HDR-BT boost. Hypofractionated WPRT is a more attractive and convenient treatment approach, and may become the new standard of care if demonstrated to be well-tolerated and effective.

**Trial registration:**

This trial was prospectively registered in ClinicalTrials.gov as NCT04197141 on December 12, 2019.

## Background

Patients with high-risk prostate cancer are at increased risk of lymph node metastasis [1]. Whole pelvis radiotherapy (WPRT) is posited to be beneficial in this population by eradicating microscopic disease outside the prostate. However, two randomized trials failed to demonstrate meaningful oncological improvements with WPRT compared to radiation to the prostate alone [2, 3]. Potential caveats may explain the lack of benefit in these trials, including use of lower doses of radiation (between 66 and 70 Gy in conventional fractionation [1.8–2 Gy per fraction]) to the prostate, which may have resulted in local progression and a second wave of metastasis; inclusion of patients at low risk of nodal disease (< 15%) thereby reducing the potential benefit of elective irradiation; and small pelvic fields without coverage of the common iliac chains.

More recently, Radiation Therapy Oncology Group (RTOG) 0924 completed accrual and is expected to definitively evaluate the role of conventionally fractionated WPRT in intermediate- and high-risk disease. However, there is already evidence that has indirectly supported the use of WPRT in high-risk prostate cancer patients. In the ASCENDE-RT trial, a WPRT + low-dose-rate brachytherapy boost strategy led to encouraging results, with 83% biochemical failure-free survival at 9 years in a cohort comprising of approximately 70% high-risk patients [4]. A recently published multicentre prospective cohort study also supports the use of WPRT in higher-risk patients, demonstrating significantly improved 5-year biochemical failure-free survival rates (84% vs. 77%, *p* = 0.001) for high-risk prostate cancer patients who received WPRT + high-dose-rate brachytherapy (HDR-BT) compared to prostate-only radiotherapy + HDR-BT [5]. Furthermore, WPRT was the superior arm in the recently presented RTOG 0534 trial [6]. In this study of patients who had biochemical failure after prostatectomy, the authors identified a 7% increase in freedom from progression in the WPRT arm compared to the prostate bed radiotherapy arm. Arguably, this benefit could be extrapolated to a cohort of high-risk patients naïve to treatment. Several retrospective series have also suggested the benefit of WPRT in prostate cancer patients [7, 8].

In parallel to studies evaluating the role of WPRT in prostate cancer, radiotherapy technique has improved in the last few decades. Technological advances, such as intensity-modulated radiotherapy (IMRT), volumetric-modulated arc therapy (VMAT) and image-guided radiotherapy, have allowed radiation oncologists to more accurately target volumes while sparing more normal tissue. In conjunction with the favourable tumour radiobiology (i.e. a low alpha/beta ratio) of prostate cancer cells, researchers have investigated the role of hypofractionated treatments (> 2 Gy per fraction) in patients with prostate cancer. A recent randomized control trial involving multiple centres showed no difference in late toxicity and cancer control between ultra-hypofractionated (≥ 5 Gy per fraction) and conventionally fractionated radiotherapy to the prostate alone [9].

The use of ultra-hypofractionation in WPRT is still in its infancy and the toxicity associated with this strategy is unclear. To our knowledge, only two small, single-arm studies have reported outcomes associated with this treatment. In both of them, a simultaneous boost was delivered to the prostate and proximal seminal vesicles. In the SATURN study [10], authors reported an acceptable toxicity profile with this approach, while authors from the FASTR trial had higher than anticipated late toxicities resulting in early trial discontinuation [11].

Currently, clinical trials such as the French SHORT trial (NCT03417336) randomize higher-risk prostate cancer patients between HDR-BT + WPRT (25 Gy in 5 fractions) versus stereotactic radiotherapy to the prostate + WPRT. However, there is no current data comparing the toxicity profile between conventionally fractionated WPRT (45 Gy in 25 fractions) versus hypofractionated WPRT (25 Gy in 5 fractions), and long-term bowel toxicity associated with this approach is unknown. Moreover, the genitourinary safety profile associated with hypofractionated WPRT plus HDR-BT boost has not yet been fully investigated.

This study aims to investigate the impact in quality of life associated with hypofractionated WPRT compared to conventionally fractionated WPRT. This information is valuable as hypofractionated WPRT is a more attractive and convenient treatment approach, and may become the new standard of care if demonstrated to be well-tolerated and effective. We hypothesize that hypofractionated WPRT is non-inferior with respect to quality of life outcomes compared to conventionally fractionated WPRT. Therefore, this study aims to provide a more rational justification for use of hypofractionated WPRT in future larger randomized trials by comparing this strategy with the current standard of care. This study will also provide an initial understanding of the toxicity profile and cancer control associated with hypofractionated WPRT and HDR-BT.

## Methods/Design

### Objectives

1. Determine if hypofractionated WPRT is non-inferior to conventionally fractionated WPRT with respect to bowel function and quality of life in prostate cancer patients treated with HDR-BT boost.
2. Assess safety, efficacy and quality of life outcomes between both treatment strategies.

### Study design

**Fig. 1:**
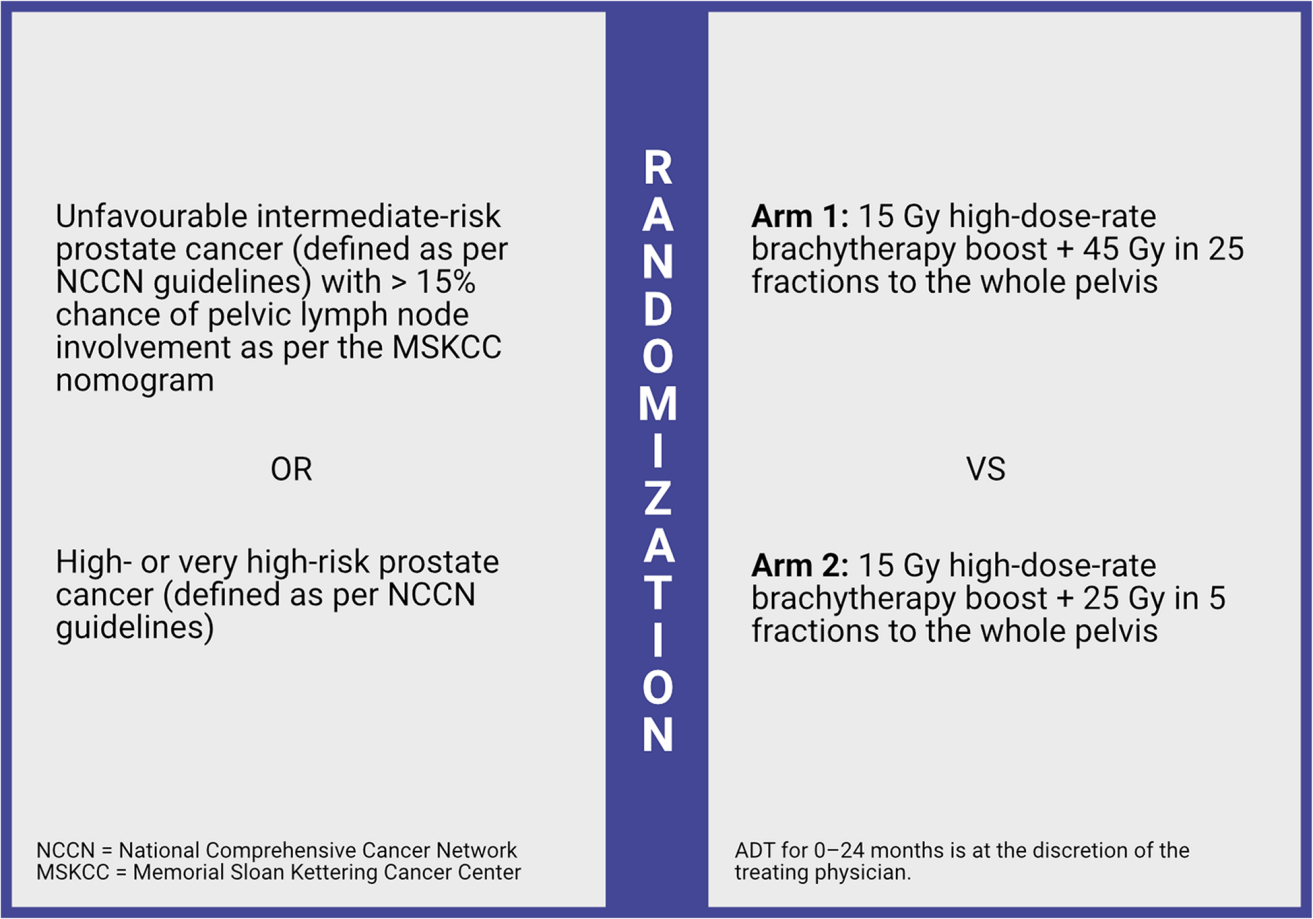
Study schema. Abbreviations: NCCN – National Comprehensive Cancer Network; MSKCC – Memorial Sloan Kettering Cancer Center

This is a phase II, open label trial, randomizing 58 unfavourable intermediate-, high- or very high-risk prostate cancer patients between HDR-BT + conventionally fractionated (45 Gy in 25 fractions) WPRT (Arm 1: 29 patients) vs. HDR-BT + hypofractionated (25 Gy in 5 fractions) WPRT (Arm 2: 29 patients) (Fig. 1).

The study will employ a 1:1 randomization between Arm 1 and Arm 2 in a permuted block design without stratification with the size of the blocks known only to the statistician.

### Endpoints

#### Primary endpoint

- Late bowel toxicity and quality of life measured using the Expanded Prostate Cancer Index Composite (EPIC) bowel function subdomain at 1 year post-treatment

#### Secondary endpoints

- Acute urinary and sexual toxicity and quality of life measured using the EPIC urinary and sexual domains at 6 weeks post-treatment
- Acute bowel toxicity and quality of life measured using the EPIC bowel domain at 6 weeks post-treatment
- Late urinary and sexual toxicity quality of life measured using the EPIC urinary and sexual domains at 1 year post-treatment
- Late bowel toxicity quality of life measured using the EPIC bowel bother subdomain at 1 year post-treatment
- International Prostate Symptom Score (IPSS), Patient-Oriented Prostate Utility Scale (PORPUS-U) and EuroQOL 5-Dimension 5-Level (EQ-5D-5L) measured at 6 weeks, 1 year and 2 years post-treatment
- Urinary, bowel and sexual toxicity measured by the Common Terminology Criteria for Adverse Events (CTCAE) version 5.0 measured at 6 weeks, 1 year and 2 years post-treatment
- Prostate-specific antigen (PSA) response rate at 4 years post-treatment
- Cost effectiveness analysis of the hypofractionated arm compared to the conventionally fractionated arm
- Freedom from local failure: Time from randomization to first local failure, or last follow-up, whichever occurs first.
- Freedom from regional failure: Time from randomization to first regional failure, or last follow-up, whichever occurs first.
- Biochemical failure-free survival: Time from randomization to biochemical failure (based on the Phoenix definition), death from any cause, or last follow-up, whichever occurs first.
- Androgen deprivation therapy (ADT)-free survival: Time from randomization to start of salvage ADT, death from any cause, or last follow-up, whichever occurs first.
- Metastasis-free survival: Time from randomization to development of metastasis, death from any cause, or last follow-up, whichever occurs first.
- Prostate cancer-free survival: Time from randomization to death attributed to prostate cancer, or last follow-up, whichever occurs first.
- Overall survival: Time from randomization to death from any cause, or last follow-up, whichever occurs first.

### Patient selection

#### Inclusion criteria

- Age 18 years or older
- Able and willing to provide informed consent
- Pathologically proven diagnosis of prostatic adenocarcinoma
- Unfavourable intermediate- (with > 15% chance of node involvement based on the Memorial Sloan Kettering Cancer Centre nomogram [12]), high- or very high-risk prostate cancer based on the National Comprehensive Cancer Network classification (PSA > 20 ng/mL; or clinical cT3a or cT3b; or Gleason score 8–10)
- Eastern Cooperative Oncology Group performance status 0–1
- No prior history of pelvic irradiation, brachytherapy, cryosurgery, high-intensity focused ultrasound, transurethral resection of the prostate or radical prostatectomy

#### Exclusion criteria

- Presence of nodal or distant metastasis, as confirmed by magnetic resonance (MR) or computed tomographic (CT) imaging of the abdomen/pelvis and bone scan within 90 days of randomization
- Plan for adjuvant docetaxel post-radiotherapy
- Serious medical comorbidities or other contraindications to HDR-BT
- Presence of inflammatory bowel disease
- Presence of connective tissue disease
- Medically unfit for general anesthesia
- Unable or unwilling to complete quality of life questionnaires

### Pre-treatment evaluation

- History and physical examination by a radiation oncologist within 6 weeks prior to randomization
- Staging investigations 90 days prior to randomization:
  - MR or CT abdomen/pelvis
  - Bone scan
- Completion of quality of life forms (EPIC, IPSS, PORPUS-U and EQ-5D-5L) on the date of enrollment
- Assessment of baseline toxicity using CTCAE version 5.0 prior to HDR-BT
- Patients undergoing treatment with ADT should have their HDR-BT scheduled for at least 2 months post-initiation of this therapy and no later than 6 months (up to 6 months of ADT prior to brachytherapy is acceptable for high-risk patients receiving long-term ADT). Patients with unfavourable intermediate-risk prostate cancer should receive HDR-BT between 2 and 4 months from ADT initiation.

### Data collection

All study data will be entered into Research Electronic Data Capture (REDCap), an electronic case report form database [13]. De-identified source documents will be uploaded directly into REDCap to support data entry.

### Treatment plan

Patients enrolled in this clinical trial will be treated with HDR-BT together with WPRT ± ADT. ADT will be at the discretion of the enrolling physician and its use should be initiated a minimum of 2 months prior to HDR-BT and no more than 4 months before HDR-BT for unfavourable intermediate-risk prostate cancer patients and no more than 6 months from the date of HDR-BT for high-risk patients receiving long-term ADT. The start date of ADT will correspond with the date of the first injection of a luteinizing hormone-releasing hormone (LHRH) agonist.

HDR-BT will be performed as previously described [14] to a dose of 15 Gy in a single fraction prescribed to the prostate. This dose and fractionation is widely used as a standard of care in the province of Ontario.

WPRT dose prescription (45 Gy in 25 fractions vs. 25 Gy in 5 fractions) will be selected according to which treatment arm the patient is randomized.

#### Androgen deprivation therapy

LHRH agonist use and total duration will be at the discretion of the enrolling physician with an acceptable range varying from 0 to 6 months for unfavourable intermediate-risk and 12–24 months for very high- or high-risk patients. Non-steroidal androgen receptor inhibitors (e.g. bicalutamide) is recommended to be used for up to 30 days prior to LHRH initiation.

#### HDR-BT planning and treatment

HDR-BT boost is a standard of care component of treatment in patients with unfavourable intermediate- and high-risk prostate cancer. This therapy will be delivered to all patients accrued in this study prior to WPRT. HDR-BT dose will consist of 15 Gy in a single fraction to the whole prostate. Patients with seminal vesicle involvement may have this organ included in the treated volume during brachytherapy at the discretion of the brachytherapist. The procedure should be performed under transrectal ultrasound-guidance. A total of three fiducial markers should be inserted in the left base, right mid-gland and left apex during the procedure. These markers will be used for prostate matching using cone beam CT and/or orthogonal kV images at the treatment unit when WPRT is being delivered.

The following dosimetric objectives should be pursued during HDR-BT:

- Prostate V_100_ ≥ 95%; V_150_ < 40%; V_200_ < 15%
- Urethra D_10_ < 118%; Urethra D_max_ < 130%
- Rectum V_80_ < 1 cc

The number of catheters inserted should vary from 10 to 18 depending on prostate volume and shape.

#### Radiotherapy planning and treatment

##### Contouring

The prostate and at least the proximal 1 cm of the seminal vesicles should be included in the WPRT clinical target volume (CTV). The whole seminal vesicles are encouraged to be included in patients with high- or very high-risk prostate cancer. Seminal vesicles should be completely covered by the CTV in patients with stage cT3b disease.

RTOG guidelines will be used for elective nodal irradiation. Obturator, pre-sacral, external and internal iliac drainages should be included in the CTV. Inclusion of the common iliac drainage is allowed and at the discretion of the treating physician.

Elective drainages, prostate and seminal vesicles should be combined to form CTV45Gy for patients randomized to Arm 1 and CTV25Gy for patients in Arm 2.

The planning target volume (PTV) is the CTV plus a 6 mm isotropic margin.

Normal critical structures that must be contoured include: bladder, rectum (from its origin at the rectosigmoid flexure until the level of the ischial tuberosities), penile bulb, bilateral femora and bowel (including sigmoid).

##### WPRT planning

Plans will be developed for IMRT or VMAT delivery techniques using an inverse planning system. If available, VMAT plans are recommended due to more conformal dose distribution and faster treatment delivery. A conformal and uniform dose distribution covering prostate, seminal vesicles and lymph nodes should be prioritized (maximum point dose inside PTV < 108%).

##### Dosimetric aims

- CTV 45Gy / 25Gy: V_100_ ≥ 99%
- PTV 45Gy / 25Gy: Ideal V_100_ ≥ 95%; Mandatory V_95_ ≥ 95%
- Conformity index (95% isodose line [42.75 Gy or 23.75 Gy] to PTV): Ideal < 1.3; Mandatory < 1.5

##### Critical organs at risk

Critical organs at risk and respective dose constraints are listed in Table 1. If the rectum and bladder constraints are not met, the PTV margin for prostate and seminal vesicles may be reduced to 5 mm.

**Table 1:** Critical organs at risk and respective dose constraints

	Arm 1: 45 Gy in 25 fractions	Arm 2: 25 Gy in 5 fractions
Rectum	V_29_ < 50%	V_18_ < 50%
	V_35_ ≤ 30%	V_20_ ≤ 30%
	D_1 cc_ ≤ 46 Gy	D_1 cc_ ≤ 26 Gy
Bladder	V_29_ < 50%	V_18_ < 50%
	V_35_ ≤ 30%	V_20_ ≤ 30%
	D_1 cc_ ≤ 47 Gy	D_1 cc_ ≤ 26 Gy
Femurs	D_10 cc_ < 45 Gy	D_10 cc_ < 25 Gy
Bowel (including sigmoid)	V_45_ < 40 cc (acceptable 60 cc) D_1 cc_ ≤ 47 Gy	V_25_ < 40 cc (acceptable 60 cc) D_1 cc_ ≤ 26 Gy
Penile bulb	Mean dose < 35 Gy	Mean dose < 20 Gy

##### Treatment delivery

Patients randomized to Arm 1 will be treated with daily fractions of WPRT from Monday to Friday. Patients randomized to Arm 2 will be treated with WPRT fractions every other day. In both arms, WPRT should begin 2–3 weeks after HDR-BT.

Daily image guidance will be performed during treatment delivery. Prostate fiducials should be initially matched. Then, the surrounding soft tissue should be evaluated including the prostate-rectum interface. Lastly, position of the pelvic nodes should be assessed.

Treatments should be delivered preferably in units with a 6-degree couch capability. Bowel preparation should be available in case large amounts of stools or gas are present inside the rectum.

##### Quality assurance

In order to ensure patient safety and effective treatment delivery, a robust quality assurance protocol is incorporated. The following requirements must be completed for each patient:

- Prior to treatment delivery, contours and plan will be peer-reviewed, either by another individual radiation oncologist or presented at genitourinary quality assurance rounds.
- All dose delivery for intensity-modulated plans (including arc-based treatments) will be reviewed before treatment by physics staff.
- Cone-beam CT and/or orthogonal kV images will be used on a daily basis to verify treatment positioning and pelvic organ filling.

### Consent process

A written informed consent will be obtained from all patients prior to radiotherapy (Additional file 1). Consents are allowed to be obtained after ADT initiation.

### Subject discontinuation and withdrawal

Subjects may voluntarily discontinue participation in the study at any time. If a subject is removed from the study, the clinical and laboratory evaluations that would have been performed at the end of the study should be obtained. If a subject is removed because of an adverse event, they should remain under medical observation as long as deemed appropriate by the treating physician.

### Follow-up evaluation and assessment of efficacy

The follow-up schedule is shown in Table 2. Day 1 of follow-up will correspond with the last day of WPRT. Additional imaging or laboratory investigations should be carried out at the discretion of the oncologist, based on findings in the history or physical examination. Additional treatment (e.g. salvage treatment with surgery or further radiotherapy) is at the discretion of the treating physicians, and will be captured in the case report form.

**Table 2:** Study follow-up schedule

	Baseline^a^	First day of WPRT	Last day of WPRT	6 weeks post-WPRT	Follow up (months)^b^						
					6	12	18	24	36	48	60
Physical Examination	X			X	X	X	X	X	X	X	X
Imaging	X										
PSA	X				X	X	X	X	X	X	X
EPIC	X	X	X	X	X	X	X	X	X	X	X
IPSS	X	X	X	X	X	X	X	X	X	X	X
CTCAE	X	X	X	X	X	X	X	X	X	X	X
Survival and disease status					X	X	X	X	X	X	X
EQ-5D and PORPUS-U	x			x		x		x			

*Abbreviations*: *WPRT* whole pelvis radiotherapy, *PSA* prostate specific antigen, *EPIC* Expanded Prostate Cancer Index Composite, *IPSS* International Prostate Symptom Score, *CTCAE* Common Terminology Criteria for Adverse Events, *EQ-5D-5L* EuroQOL 5-Dimension 5-Level, *PORPUS-U* Patient-Oriented Prostate Utility Scale

^a^Baseline assessments to be completed prior to high-dose-rate brachytherapy

^b^Follow-up schedule determined based on day of last WPRT treatment

### Statistics and sample size calculation

#### Sample size

Sample size calculation was performed by using a two-sided two-sample t-test for non-inferiority. Treatment strategies will be considered non-inferior if the difference between groups remains within the minimum clinical significance (5 points) for the bowel function subdomain based on EPIC. With an alpha = 0.05 and a standard deviation = 5, 23 patients in each arm are needed for a power = 0.90. Since the London Regional Cancer Program covers a large catchment area, 20% are estimated to be lost to follow-up, therefore 29 patients in each arm (29 × 0.8 = 23.2) or 58 total patients will be required.

#### Analysis plan

Patients will be analyzed in the groups to which they are assigned (intention-to-treat).

Comparisons between treatment arms for acute and late urinary, bowel and sexual quality of life end points will be performed using the two-sample t-test for non-inferiority. Quality of life data is anticipated to be normally distributed. In the event such data is not normally distributed, the Wilcoxon rank sum test for non-inferiority will be substituted as appropriate. Similarly, IPSS data is anticipated to be normally distributed and will follow similar methodology. Differences in urinary, bowel and sexual toxicity and PSA response rate at 4 years post-treatment between treatment arms will be compared using the Chi–square test or Fisher’s exact test as appropriate. Time-to-event end points (e.g. overall survival) will be compared between treatment arms using Kaplan-Meier estimates and the log-rank test.

In the event the non-inferiority criteria is met for the primary end point (late bowel function quality of life based on EPIC), a secondary analysis to investigate superiority of the experimental arm will take place. This will be performed using the two-sample t-test (or Wilcoxon rank sum test as appropriate as similarly discussed above). A 5-point difference in scores will be considered a clinically meaningful change.

For each quality of life domain and IPSS data, linear mixed effects models will be generated to test for differences between treatment arms over time. Cox proportional hazards univariable and multivariable regression models will be used to identify baseline factors predictive of time-to-event end points (e.g. overall survival).

#### Data and safety monitoring committee

The Data and Safety Monitoring Committee (DSMC) will act in an advisory capacity to the principal investigator to monitor patient safety. The DSMC will meet every 6 months after study initiation.

#### Interim analysis

An interim analysis will be conducted once 29 patients have been accrued and followed for 1-year. For this analysis, the DSMC will be blinded to the identity of each treatment arm. If the standard deviations of the quality of life scores are substantially different than estimated in the sample size calculation, the DSMC can recommend increasing the target accrual in order to maintain the study statistical power.

### Confidentiality of subject records

Study participant personal health information will be kept confidential. All study records identify the participants by initials and a unique identification number. A master list that links participants to their record numbers will be kept confidential by a data coordinator. Access to identifying or personal health information will only be permitted for those with involved with direct subject management and data monitors. No names will be used in any public report of the study.

### Protocol amendments

The trial protocol will be amended only by the approval of the principal investigator (current version: 1.4 on July 14, 2020). It is the responsibility of the principal investigator to disseminate amendments to co-investigators, research boards and trial registries. Authorship of the trial abstract and manuscript will be decided by the principal investigator at the time of submission.

## Discussion

This study aims to investigate quality of life outcomes associated with hypofractionated WPRT by comparing them to conventionally fractionated WPRT in prostate cancer patients receiving brachytherapy boost. This is a pivotal question as the role of WPRT in patients with higher risk prostate cancer is becoming more established and preliminary studies indicate that hypofractionated WPRT is well-tolerated.

The current literature assessing hypofractionated WPRT in prostate cancer patients is scarce. Previously, trials like SATURN and FASTR have investigated the role of WPRT together with a concomitant stereotactic ablative radiotherapy (SABR) boost to the prostate [10, 11]. However, these were single-arm trials and therefore a direct comparison to the standard of care was not performed. In contrast, the HOPE trial investigates the tolerability of ultra-hypofractionated WPRT by directly comparing this treatment strategy to a conventionally fractionated regimen and employing validated patient reported outcome measures to detect differences in bowel, urinary and sexual function. Assuming an alpha/beta of 3, the two schedules are anticipated to be similar in toxicity with a biologically equivalent dose of 72 and 66.67 Gy for the conventionally and ultra-hypofractionated schemes respectively.

This trial also differs from the other studies because uses HDR-BT boost instead of SABR. At least three randomized trials have shown that a brachytherapy boost reduces, at a minimum, biochemical relapses when compared to external beam radiotherapy (EBRT) in conventional or dose-escalated regimens [4, 15, 16]. Brachytherapy boost also reduces the dose to the prostate and seminal vesicles given through EBRT and therefore potentially reduces the amount of dose received by the anterior wall of the rectum and the long-term toxicities associated with this treatment [17].

Recently, a study involving a 15 Gy HDR-BT boost to the prostate followed by 23.9 Gy in 5 daily fractions of EBRT (4.78 Gy per fraction) to the prostate and proximal seminal vesicles suggested a high disease control rate and good tolerability associated with this regimen [18]. In this analysis, only intermediate-risk prostate cancer patients were enrolled and WPRT was not offered. Urinary function based on EPIC questionnaires was stable throughout the follow-up period suggesting good urinary tolerability. Nevertheless, the lack of a control group is a weakness of this study.

To the best of our knowledge, this is the first study to compare hypofractionated WPRT to conventionally fractionated WPRT in the HDR-BT boost setting. A strength of this study is its randomized design, which avoids selection bias inherent to retrospective studies or single-arm prospective studies. Further, this trial primarily investigates impact on quality of life endpoints associated with hypofractionated WPRT through a non-inferiority study design and by using a validated patient-reported outcome questionnaire. These questionnaires are known to better represent symptomatic adverse reactions experienced by patients receiving WPRT when compared to physician-reported endpoints [19].

## Supplementary information

**Additional file 1.**

## Data Availability

The datasets used and/or analysed during the current study are available from the corresponding author on reasonable request.

## Abbreviations

ADT: Androgen deprivation therapy
CT: Computed tomography
CTCAE: Common Terminology Criteria for Adverse Events
CTV: Clinical target volume
DSMC: Data and Safety Monitoring Committee
EBRT: External beam radiotherapy
EPIC: Expanded Prostate Cancer Index
EQ-5D-5L: EuroQOL 5-Dimension 5-Level
HDR-BT: High-dose-rate brachytherapy
IMRT: Intensity-modulated radiotherapy
IPSS: International Prostate Symptom Score
LHRH: Luteinizing hormone-releasing hormone
MR: Magnetic resonance
PORPUS-U: Patient-Oriented Prostate Utility Scale
PSA: Prostate-specific antigen
PTV: Planning target volume
REDCap: Research Electronic Data Capture
RTOG: Radiation Therapy Oncology Group
SABR: Stereotactic ablative radiotherapy
VMAT: Volumetric modulated arc therapy
WPRT: Whole pelvis radiotherapy
