# Supplementary material for: Is hypofractionated whole pelvis radiotherapy (WPRT) as well tolerated as conventionally fractionated WPRT in prostate cancer patients? The HOPE trial
Source: BMC Cancer. 2020 Oct 9;20:978. doi: 10.1186/s12885-020-07490-0 (PMC7547418; doi:10.1186/s12885-020-07490-0)
Supplement: Supplementary file 1 — Additional file 1. [file 12885_2020_7490_MOESM1_ESM.pdf]

London Health Sciences Centre
London Regional Cancer Program
800 Commissioners Road East
PO Box 5010, STN B
London, Ontario N6A 5W9
Tel 519.685-8500

Letter of Information and Consent

Is hypofractionated whole-pelvis radiotherapy (WPRT) as well tolerated as conventionally-fractionated WPRT in prostate cancer patients (The HOPE-Trial)

Principal Investigator: Dr. Lucas C. Mendez
Radiation Oncology, London Regional Cancer Program
519-685-8650

Sponsor: Dr. Lucas C. Mendez

Study Funders: Academic Medical Organization of Southwestern Ontario
Abbvie-Caro Uro-Oncologic Radiation Awards – Canadian Association of Radiation Oncology

Emergency Contact Number (24 hours / 7 days a week): If a medical emergency arises, proceed to your local Emergency Department. The emergency physician can contact the oncologist on call at 519-685-8500 if required.

Non-Emergency contact numbers are at the end of this document under Contacts.

Introduction

You are being invited to participate in this study, because you have been diagnosed with prostate cancer that requires treatment with brachytherapy (internal radiation) followed by external radiotherapy to the pelvis. Please read the following information carefully and discuss it with your family and/or family doctor, if you wish. If you decide to participate, you will be asked to sign the consent form before you can be enrolled in the study.

Background and Purpose

The purpose of this research study is to determine if 5 (five) fractions of external radiotherapy with higher radiation doses per fraction to the pelvis leads to similar results to the standard of care external radiotherapy treatment that is comprised of 25 fractions of external radiotherapy with lower radiation doses per fraction to the pelvis.

The combination of brachytherapy plus external radiotherapy has been shown to improve outcomes in patients with unfavorable-intermediate and high-risk prostate cancers. Currently, brachytherapy is delivered in a single fraction and as an outpatient procedure.

Recently, researchers have been interested in reducing the external radiation time length to increase treatment convenience and reduce costs. Nevertheless, a proper comparison between this newly proposed treatment (in 5 fractions) and the current standard of care (in 25 fractions) has not yet been investigated. This study aims to evaluate this question.

Study Participant's Initials: _____.

How many people will take part in this study?

This study will run for up to 5 years and we will be enrolling a total of 58 men, all of whom will be patients at the London Regional Cancer Program at London Health Sciences Centre in London, Ontario.

Study Procedures

Assignment to a group

If you agree to participate, you will be "randomized" into one of the groups described below. Randomization means that you are put into a group by chance (like flipping a coin). There is no way to predict which group you will be assigned to. You will have an equal, 50/50, chance of being placed in either group. Neither you, the study staff, nor the study doctors can choose what group you will be in.

You will be told which group you are in.

Study Treatment

All participants will receive standard brachytherapy treatment prior to assigned external radiation treatment. The procedure will take approximately 3 hours and is performed under general anesthesia.

Group 1 – Standard of Care Conventional Treatment - Brachytherapy plus 25 fractions of external radiation to the pelvis

Radiation treatment will be delivered Monday to Friday for 5 weeks and will begin 2 to 3 weeks following the brachytherapy treatment. Each treatment will take approximately 10-15 minutes.

Group 2 – Hypofractionated Treatment - Brachytherapy plus 5 fractions of external radiation to the pelvis

Radiation treatment will be delivered every other day for 5 treatments and will begin 2 to 3 weeks following the brachytherapy treatment. Each treatment will take approximately 10-15 minutes.

Questionnaires

You will be provided with 4 questionnaires to complete before starting this study, and then the first and last day of treatment, 6 weeks after treatment for 2 years, then yearly for 3 years. The purpose of the questionnaire is to understand how your treatment and illness affects your quality of life. Each questionnaire will take about 10 minutes to complete.

The information you provide is for research purposes only. Some of the questions are personal. You can choose not to answer if you wish.

Even though you may have provided information on a questionnaire, these responses will not be reviewed by your health care team or study team at your clinic visit, the questionnaires will be reviewed during analysis at the end of the study. If you wish them to know this information please bring it to their attention.

The following table lists the study procedures and when they will occur during the study.

	**Baseline[1]**	**First day of WPRT**	**Last Day of WPRT**	**6 weeks post WPRT**	**Follow up (months)[2]**						
					**6**	**12**	**18**	**24**	**36**	**48**	**60**
**Physical Exam**	X			X	X	X	X	X	X	X	X
**Imaging**	X										
**PSA**	X				X	X	X	X	X	X	X
**EPIC**	X	X	X	X	X	X	X	X	X	X	X
**IPSS**	X	X	X	X	X	X	X	X	X	X	X
**CTCAE Assessment**	X	X	X	X	X	X	X	X	X	X	X
**Survival and disease status**					X	X	X	X	X	X	X
**EQ-5D and PORPUS-U**	x			x		x		x			

1. Baseline assessments to be completed prior to HDR-BT
2. Follow up schedule determined based on day of last WPRT treatment

Alternatives to Study Participation

You do not have to take part in this study in order to receive treatment/care. Other options may include, but are not limited to:

- Standard of care treatment comprised of brachytherapy followed by external radiation to the pelvis in 25 fractions (similar to the standard arm of this study).

Please talk to your study doctor or usual cancer doctor about the known benefits and risks of these other options before you decide to take part in this study. Your usual cancer doctor can also discuss with you what will happen if you decide not to undertake any treatment at this time.

Responsibilities

If you choose to participate in this study, you will be expected to:

- Tell your study doctor about your current medical conditions;

- Tell your study doctor about all prescription and non-prescription medications and supplements, including vitamins and herbals, and check with your study doctor before starting, stopping or changing any of these. This is for your safety as these may interact with the treatment you receive on this study;
- Tell your study doctor if you are thinking about participating on another research study;

Length of Participation

Your total treatment time will last about 8 weeks if you are randomized to the standard arm and 5 weeks if you are randomized to the experimental arm.

The study will continue for up to 5 years or until your study doctor decides to stop the study. You will be asked to come back to the clinic/hospital for standard follow-up visits according to your hospital's guidelines. Study staff will collect study data on your health status for 5 years.

Early End to Participation

Your participation in the study may be stopped early, for reasons such as:

- The treatment does not work for you and your cancer gets worse.
- You are unable to tolerate the study treatment.
- You are unable to complete all required study procedures.
- New information shows that the study treatment is no longer in your best interest.
- Your study doctor no longer feels this is the best treatment for you.
- The study doctor decides to stop the study.

Risks and Discomforts to You If You Participate In This Study

Participating in this study will put you at risk for the side effects listed below. You should discuss these with your study doctor. As with any treatment additional unexpected and sometimes serious side effects are a possibility. Your study doctor will monitor you closely to see if you have side effects.

Preliminary studies have indicated that external radiation to the pelvis in 5 fractions seems to be well tolerated, but a formal comparison to the standard of care (25 fractions) is lacking. In a Canadian leading experience involving 30 patients (SATURN-Trial), a similar treatment external radiation treatment was well tolerated after more than 2 years of follow-up time. Participants receiving external radiotherapy to the pelvis may experience fatigue, lack of energy, urinary symptoms (e.g. urinary frequency, burning sensation to void, slow stream and urgency) and bowel symptoms (e.g. loose stools or diarrhea). At this moment, it is unknown if participants treated with 5 fractions (experimental arm) will have more or less intense side effects than participants treated with 25 fractions. In fact, this is the question that this study is trying to answer.

Potential Benefits

There is no guarantee that you will benefit from participating in this study. Participants randomized to the experimental arm may benefit from a less intense time commitment (more convenience).

Confidentiality:

Records identifying you at this centre will be kept confidential and, to the extent permitted by the applicable laws, will not be disclosed or made publicly available, except as described in this letter of information and consent document.

Authorized representatives of the following organizations may look at your original (identifiable) medical/clinical study records at the site where these records are held, for quality assurance (to check that the information collected for the study is correct and follows proper laws and guidelines):

- Clinical Cancer Research Unit (LHSC/HHSC), the research group coordinating this study
- Dr. Lucas Mendez, the sponsor of the study
- Western University's Research Ethics Board, which oversees the ethical conduct of this study in your clinic/hospital
- The Quality Assurance and Education Officers from Lawson Health Research Institute (Lawson) may audit this research study for quality assurance purposes.

All of the organizations listed in the above confidentiality section are required to have strict policies and procedures to keep the information they see or receive about you confidential, except where disclosure may be required by law. The study doctor will ensure that any personal health information collected for this study is kept in a secure and confidential location at the London Regional Cancer Program, London Health Sciences Centre for 25 years as required by law. There are federal and provincial laws that these organizations must comply with to protect your privacy.

If the results of this study are published, your identity will remain confidential. It is expected that the information collected during this study will be used in analyses and will be published/ presented to the scientific community at meetings and in journals.

Even though the likelihood that someone may identify you from the study data is very small, it can never be completely eliminated.

Your family doctor will be informed that you are taking part in a study so that your study doctor and your family doctor can provide proper medical care.

A copy of this signed and dated letter of information and consent form may be included in your health record/hospital chart. Your de-identified data from this study may be used for other research studies. If your study data is shared with other researchers, information that links your study data directly to you will not be shared.

Registration of Clinical Trials

A description of this clinical trial will be available on http://www.clinicaltrials.gov. This Web site will not include information that can identify you. At most, the Web site will include a summary of

the results. You can search this website at any time. This description is mandatory for every clinical trial worldwide.

Costs

The study treatment will be provided to you free of charge while you are participating in this study. The costs of your medical treatment will be paid for by your provincial medical plan to the extent that such coverage is available. There may be extra costs that are not covered by your medical plan that you will have to pay yourself; some examples may be physiotherapy or certain pain medications.

Taking part in this study may result in added costs to you (i.e. transportation, parking meals, or unpaid leave from work). You may have to pay for medication prescribed to treat or prevent side effects, and you may have to visit the hospital more often than if you were not participating in this study.

Compensation

You will not be paid for taking part in this study.

Should you be required to visit the centre more frequently than if you were receiving standard treatment (not participating in this study), you will be reimbursed for your parking expenses for these extra visits.

It is possible that the research conducted using your study data may eventually lead to the development of new diagnostic tests, new treatments or other commercial products. There are no plans to provide payment to you if this happens.

In the case of research-related side effects or injury, medical care will be provided by your doctor or you will be referred for appropriate medical care at no cost to you.

Rights

You will be told, in a timely manner, about new information that may be relevant to your willingness to stay in this study. If you decide to stop participating in the study or if your participation has been stopped, your study doctor will discuss other options with you and continue to treat you with the best means available.

You may withdraw your permission to use your personal health information for this study at any time by letting the study doctor know. However, this would also mean that you withdraw from the study. Your study data that was recorded before you withdrew will be used but no information will be collected or sent to the study sponsor after you withdraw your permission. Your rights to privacy are legally protected by federal and provincial laws that require safeguards to ensure that your privacy is respected.

By signing this form you do not give up any of your legal rights against the investigators, sponsor or involved institutions for compensation, nor does this form relieve the investigators, sponsor or involved institutions of their legal and professional responsibilities.

You will be given a copy of this signed and dated letter of information and consent form prior to participating in this study.

Conflict of Interest

This project is receiving funds from Academic Medical Organization of Southwestern Ontario (AMOSO) as well as from ACURA-CARO (Abbvie-Caro Uro-Oncologic Radiation Awards – Canadian Association of Radiation Oncology) to help offset the costs of conducting this research. The researchers at this centre will not receive any direct benefit for conducting this study. The doctor treating you also may be the doctor in charge of the study.

Contacts

If you have questions about taking part in this study, or if you suffer a research-related injury, you should talk to your study doctor. Or, you can meet with the doctor who is in charge of the study at this institution. That person is:

Dr. Lucas Mendez (519) 685-8650

If you have questions about your rights as a participant or about ethical issues related to this study, you can talk to someone who is not involved in the study at all. Please contact Patient Relations Office at London Health Sciences Centre at (519) 685-8500 ext. 52036 or access the online form at: https://apps.lhsc.on.ca/?q=forms/patient-experience-contact-form

London Health Sciences Centre
London Regional Cancer Program
800 Commissioners Road East
PO Box 5010, STN B
London, Ontario N6A 5W9
Tel 519.685-8500

Consent Form

Is hypofractionated whole-pelvis radiotherapy (WPRT) as well tolerated as conventionally-fractionated WPRT in prostate cancer patients (The HOPE-Trial)

I have read the Letter of Information, have had the nature of the study explained to me and I agree to participate. All questions have been answered to my satisfaction. I consent to the study team to use and share my health information as described in this form. I will receive a copy of the signed letter of information and this consent form.

To be signed and dated by the study participant

________________________________ __________________________
Signature of study participant | Date of Signature

____
Printed name of study participant (BLOCK CAPITALS)

________________________________ __________________________
Signature of person conducting the informed consent discussion | Date of Signature

____
Printed name of person conducting the informed consent discussion (BLOCK CAPITALS)

________________________________ __________________________
Signature of impartial witness | Date of Signature

____
Printed name of impartial witness (BLOCK CAPITALS)

Was the participant assisted during the consent process? ☐ YES ☐ NO

If YES, please check the relevant box and complete the signature space below:
The person signing below acted as a translator for the participant during the consent process and attests that the study as set out in this form was accurately translated and has had any questions answered.

________________________	________________	____________________
Print Name of Translator	Signature	Date (DD-MMM-YYYY)

____
Language
